# Combined RAS Modulation: The Effect on Plasma and Tissue Angiotensin Peptide Levels

**DOI:** 10.33549/physiolres.935764

**Published:** 2025-12-01

**Authors:** Ludovit PAULIS, Romana RAJKOVICOVA, Kristina REPOVA, Gabriela GUBO, Andrej BARTA, Marko POGLITSCH, Oliver DOMENIG, Natalia ANDELOVA, Miroslav FERKO, Olga PECHANOVA, Fedor SIMKO

**Affiliations:** 1 Institute of Pathophysiology, Faculty of Medicine, Comenius University, Bratislava, Slovak Republic; 2 Centre for Experimental Medicine, v.v.i., Slovak Academy of Sciences, Bratislava, Slovak Republic; 3 Attoquant Diagnostics, Vienna, Austria

**Keywords:** Renin, angiotensin system, Angiotensin peptides, Angiotensin II Type 1 Receptor Blockers, Angiotensin-Converting Enzyme Inhibitors, Renin Inhibitors

## Abstract

Combined renin–angiotensin system (RAS) inhibition can enhance blood pressure control but has not improved clinical outcomes, underscoring the importance of complex changes in angiotensin peptide profiles in combined RAS blockade. We investigated hemodynamics and circulating and tissue angiotensin peptide profile in spontaneously hypertensive rats (SHR) treated with lisinopril, olmesartan and aliskiren and their dual combinations. SHR exhibited hypertension and left ventricular hypertrophy along with reduced circulating Ang I, Ang II, and Ang 1–7. Lisinopril produced the most pronounced antihypertensive effects, with additional reduction when combined with olmesartan or aliskiren. In contrast, aliskiren – either alone or in combination – had only modest effects in this low-RAS setting. The morphological changes of the myocardium largely mirrored the blood pressure responses across treatment groups, reinforcing the hemodynamic basis of structural remodeling in SHR. Lisinopril and olmesartan markedly increased Ang I and Ang 1–7, but lisinopril suppressed Ang II while olmesartan increased Ang II. Aliskiren further reduced Ang II and Ang 1–7. Across treatment strategies, dual RAS blockade frequently decreased both renal and circulating Ang 1–7 despite greater hemodynamic efficacy. Tissue analyses revealed minimal intrinsic Ang II synthesis in the left ventricle, consistent with AT_1_-dependent uptake of circulating Ang II, while renal peptide profiles indicated some local enzymatic activity with differential reliance on ACE and neprilysin. Our results advocate a cautious, mechanism-aware approach to combination RAS blockade and support therapeutic strategies that balance blood pressure lowering with preservation of the Ang 1–7 axis.

## Introduction

Blockade of the renin–angiotensin system (RAS) is a cornerstone of cardiovascular risk reduction [1]. Therapeutic interventions in the RAS are possible at several levels, including direct renin inhibition (RI) reducing Ang I generation; angiotensin-converting enzyme (ACE) inhibitors (ACEi); and angiotensin receptor blocker (ARBs) [2]. These interventions exert distinct and sometimes opposing effects on upstream (Ang I) and downstream (Ang II, Ang 1–7) peptide concentrations, due to their different positions within the enzymatic cascade [1].

Although dual RAS blockade frequently yields additional blood pressure reduction to monotherapy, clinical outcomes have not supported its routine use [3]. Notably, the ONTARGET trial in high-risk patients displayed more adverse events (such as hypotension, syncope, renal impairment, hyperkalemia) in the combined arm while no improvement in the primary or secondary efficacy end-points was observed [4]. Similarly, The ALTITUDE trial, which tested the RI, aliskiren, in diabetic patients with renal disease needed to be halted prematurely due to increased incidence of adverse events (slight numeric increase in stroke rates and significant increase in hyperkalemia and hypotension) in the aliskiren arm (on the background of ACE inhibition or ARB treatment) [5].

These studies have generated several hypotheses for their outcomes. It was hypothesized that lower blood pressure in the combination arm might have contributed to the increased incidence of renal end-points in the combination arm of the ONTARGET trial [4] and that the low blood pressure at baseline might be responsible for the renal dysfunction observed in the aliskiren arm of the ALTITUDE trial [5]. Indeed a meta-analysis demonstrated that dual therapy was associated with 66 % higher risk of hypotension [6]. However, with regard to the primary end-point, the ARB+ACEi combination in OTARGET performed worst in patients with baseline BP 134–150 mmHg, with better outcomes in patients with BP less than 134 mm Hg [4]. Moreover, the blood pressure difference in the combined arm was negligible in both the ONTARGET and ALTITUTDE trial [4,5].

Further hypotheses suggested the excess renal adverse events observed with dual RAS blockade not primarily attributable to hypotension. First, the combined ACEi/ARB or ACEi/RI therapy might cause marked efferent arteriolar vasodilation, reducing glomerular capillary pressure and predisposing to abrupt declines in GFR [7]. Second, suppression of both Ang II–dependent and aldosterone-dependent pathways impairs renal potassium and sodium handling, thereby increasing the risk of hyperkalemia and volume-related hemodynamic stress [8]. Third, dual blockade produces a deeper reduction in intrarenal RAS activity and renin secretion, which may diminish the kidney’s adaptive responses to reduced perfusion pressure [9].

Still, in-depth mechanistic explanations remain limited, particularly regarding how different forms of RAS inhibition reshape the circulating and tissue angiotensin peptide landscape. Comparative data on how single versus combined RAS inhibition affects individual downstream peptides – especially Ang I, Ang II, and the potentially protective Ang 1–7 – are sparse. These peptides are potent regulators of several mechanisms implicated in the adverse effects of dual blockade, including glomerular hemodynamics, tubular sodium and potassium handling, and inflammation. Moreover, tissue-specific RAS systems (particularly in the kidney) may operate differently from the circulating RAS due to differences in local ACE/ACE2 expression, peptide uptake, and enzymatic degradation pathways [10,11]. Understanding these interactions requires direct measurement of peptides across both plasma and tissues.

Therefore, in our study, we aimed to determine how single and combined RAS blockade affects (i) systemic hemodynamics, (ii) myocardial hypertrophy, (iii) renin activity, and (iv) the distribution of key angiotensin peptides (Ang I, Ang II, Ang 1–7) in plasma and tissues (left ventricle, kidney) in spontaneously hypertensive rats (SHR) versus normotensive Wistar-Kyoto (WKY) controls.

## Methods

### Animals and treatment

Male 10-week old Wistar Kyoto rats (WKY) and spontaneously hypertensive rats (SHR) (Janvier, Le Genest-St-Isle, France) were used in the experiment. WKY (n=5) served as normotensive controls. SHR were randomly assigned into 7 groups treated with either vehicle (SHR, n=6), 15 mg/kg/day lisinopril (LIS, n=5), 10 mg/kg/day olmesartan (OLM, n=6), 75 mg/kg/day aliskiren (ALI, n=6), or their combinations: LIS+OLM (n=5), LIS+ALI (n=5) and OLM+ALI (n=6) for 4 weeks. Medications were applied orally by pipette once daily between 08:30 a.m. and 10:00 a.m. Normotensive and hypertensive control animals were sham-pipetted with an equal volume of vehicle (*aqua ad injectabilia*). Animals were housed under standard laboratory conditions (temperature 23 ºC, 12-h light-dark cycle), they were fed a standard pellet diet (1% NaCl) and drank tap water ad libitum. All procedures and experimental protocols, which were approved by the State Veterinary and Food Administration of the Slovak Republic conformed to Good Publication Practice in Physiology [12].

### Non-invasive blood pressure measurement

Systolic blood pressure (SBP) and heart rate (HR) were measured weekly using tail-cuff plethysmography (ADInstruments, Spechbach, Germany) after a two-week habituation period. Each measurement consisted of at least five consecutive inflation cycles, and the mean of the last three stable readings was used for analysis. Measurements were performed in a temperature-controlled environment (32–34 °C) to promote tail blood flow.

### Echocardiography

After four weeks of treatment, transthoracic echocardiography was performed by an experienced echocardiographer blinded to the group identity. A 14-MHz matrix probe (M12L) coupled with a GE Medical Vivid 7 Dimension System (GE Medical Systems CZ Ltd., Prague, Czech Republic) was used. Anesthesia was maintained by applying a 2.5% inspiratory concentration of isoflurane at a flow rate of 2 L/min through a sealed nose cone during spontaneous breathing. The rat was placed in the supine position on a warming pad (38 °C) and the thoracic wall was shaved. The heart rate and body temperature were monitored continuously. Measurements (IVSd/s, PWTd/s, LVIDd/s) were obtained from M-mode tracings in the long-axis view, and values were averaged from three consecutive cardiac cycles. Left ventricular end-diastolic volume (EDV=0.5*7/(2.4+LVIDd) *LVIDd^3), end-systolic volume (ESV=7/(2.4+LVIDs)* LVIDs^3), stroke volume (SV=EDV-ESV), and fractional shortening (FS=(LVIDd–LVIDs)/LVIDd ×100) were calculated. EDV Teichholz formula was applied with correction factor for rodent echocardiography [13,14]. LV mass (LVM = 0.8*(ρ*((LVIDd+IVSd+PWTd)^3-(LVIDd)^3))+0.6) was calculated assuming specific myocardial mass ρ=1.04 mg/ml.

### Sampling and heart weight

After four weeks of treatment, the rats were by decapitation under deep isoflurane anesthesia. The heart and kidneys were rapidly excised, rinsed in ice-cold saline, blotted dry, and weighed. Heart weight (HW) and tibia length (TL) were determined and HW/TL ratio was calculated. Cross-sectional sample of the myocardium was processed for morphometric analysis and further samples of the left ventricle and of the kidney were processed for angiotensin peptide determination as described below. Blood samples were collected from the abdominal aorta during euthanasia and processed for subsequent angiotensin analysis as described below. Left ventricular and renal samples for peptide analysis and the blood samples were snap-frozen in liquid nitrogen and stored at –80 °C.

### Left ventricle morphometry

Cross-sectional myocardial segments at the level of the papillary muscles were fixed in 4% paraformaldehyde for 24 h, embedded in paraffin, and sectioned at 5 μm and stained with haematoxylin and eosin. Because the hearts were not perfusion-fixed, geometric indices were interpreted accordingly.

Morphometric analysis was performed by an investigator blinded to group identity using a Nikon-119 microscope with a CCD camera and ImageJ software (National Institutes of Health, USA).

The left ventricle wall thickness (LVWT, five measurements per sample) and septal wall thickness (SWT, 3 measurements per sample) was determined at 400x magnification and the inner circumference (IC) was determined with 50x magnification. The LV inner diameter, LVID=IC/π and median LVWT and SWT were calculated. Finally, the LV mass (LVM) was calculated assuming spherical LV shape (LVM=ρ*((SWT + LVWT + LVID)^3 - (LVID)^3)) and specific myocardial mass ρ=1.04 mg/ml.

### Angiotensin peptides quantification

Angiotensin peptides were quantified in plasma (snapshot and equilibrium) and in LV and kidney homogenates (equilibrium) using LC–MS/MS as described previously [15,16].

Snapshot plasma samples were collected into tubes preloaded with protease inhibitor cocktail (Attoquant, Vienna), centrifuged at 2000×g for 15 min at 4 °C, and stored at –80 °C. For equilibrium measurements, plasma or homogenized tissue samples were incubated at 37 °C for 30 min to allow endogenous enzymatic activity before stabilization.

Samples were spiked with isotopically labeled internal standards (200 pg/mL) before C18 solid-phase extraction and LC–MS/MS analysis (reversed-phase analytical column (Acquity UPLC® C18, Waters Corp., Milford, MA, USA) operating in line with a XEVO TQ-S triple quadrupole mass spectrometer (Waters Corp.) in MRM mode). Internal standards were used to correct for peptide recovery of the sample preparation procedure for each angiotensin metabolite in each individual sample. Ang peptide concentrations were calculated considering the corresponding response factors determined in appropriate calibration curves in the original sample matrix, on the condition that integrated signals exceeded a signal-to-noise ratio of 10.

The equilibrium Ang II/Ang I ratio and Ang 1–7/Ang II ratio were used as surrogates for angiotensin-converting enzyme (ACE) and angiotensin-converting enzyme 2 (ACE2) activities, respectively.

### Statistics

Statistical analyses were performed using one-way ANOVA with Tukey’s post-hoc test for multiple comparisons (GraphPad InStat 3.06). Normality was assessed using the Kolmogorov–Smirnov test, and homogeneity of variance was evaluated using Bartlett’s test. Pre-specified post-hoc comparisons included: (i) WKY vs all groups, (ii) SHR vs all groups, (iii) monotherapies vs each other, and (iv) each combination vs its component monotherapies.

Data are expressed as mean ± SEM, and statistical significance was defined as p<0.05.

## Results

### Systolic blood pressure and heart weight

Compared to WKY, SHR exhibited significantly elevated SBP and a trend toward higher HR. Lisinopril and olmesartan significantly lowered SBP, whereas aliskiren did not differ from untreated SHR. Both lisinopril-based combinations (LIS+OLM, LIS+ALI) produced robust SBP reductions, reaching values that were numerically below WKY. SBP in OLM+ALI was numerically similar to OLM and significantly reduced compared to ALI but not compared to SHR (Table 1).

Absolute HW and relative HW (HW/TL) were increased in SHR compared to WKY. Only LIS and combinations of lisinopril (LIS+OLM and LIS+ALI) significantly reduced HW/TL. Moreover, lisinopril addition to olmesartan or aliskiren achieved superior reduction compared to the respective monotherapy (OLM or ALI, resp.). The HW/TL in OLM+ALI was numerically similar to OLM and numerically lower compared to ALI (Table 1).

### Histomorphometry

Compared to WKY, the geometry of the LV in SHR, assessed by histomorphometry, was characterized by numerically increased LVWT and numerically reduced LVID producing numerical increase in absolute (LVM) and relative (LVM/TL) calculated mass (Table 1).

Although no significant changes in LVM/TL were observed, LIS and ALI showed values numerically similar to WKY and the combinations of lisinopril (LIS+OLM and LIS+ALI) values even lower compared to WKY. These two groups were characterized by significant LVWT reductions compared to SHR (Table 1).

### Echocardiography

Echocardiographically, SHR displayed mild increases in diastolic wall thickness, EDV, and ESV, accompanied by non-significant reductions in SV and FS compared to WKY. The absolute LVM and relative LVM (LVM/TL) were increased only numerically compared to WKY (Table 2).

ANOVA revealed significant treatment effects for LVIDs/d, ESV, EDV, SV and LVM/TL; however, post-hoc comparisons were largely non-significant except for OLM+ALI, which showed higher LVIDs/d, ESV, and EDV compared to both SHR and WKY. LVM/TL was numerically highest in OLM+ALI and lowest in both lisinopril treated combinations (LIS+OLM and LIS+ALI) (Table 2).

### Circulating angiotensin profile

**Fig. 1:**
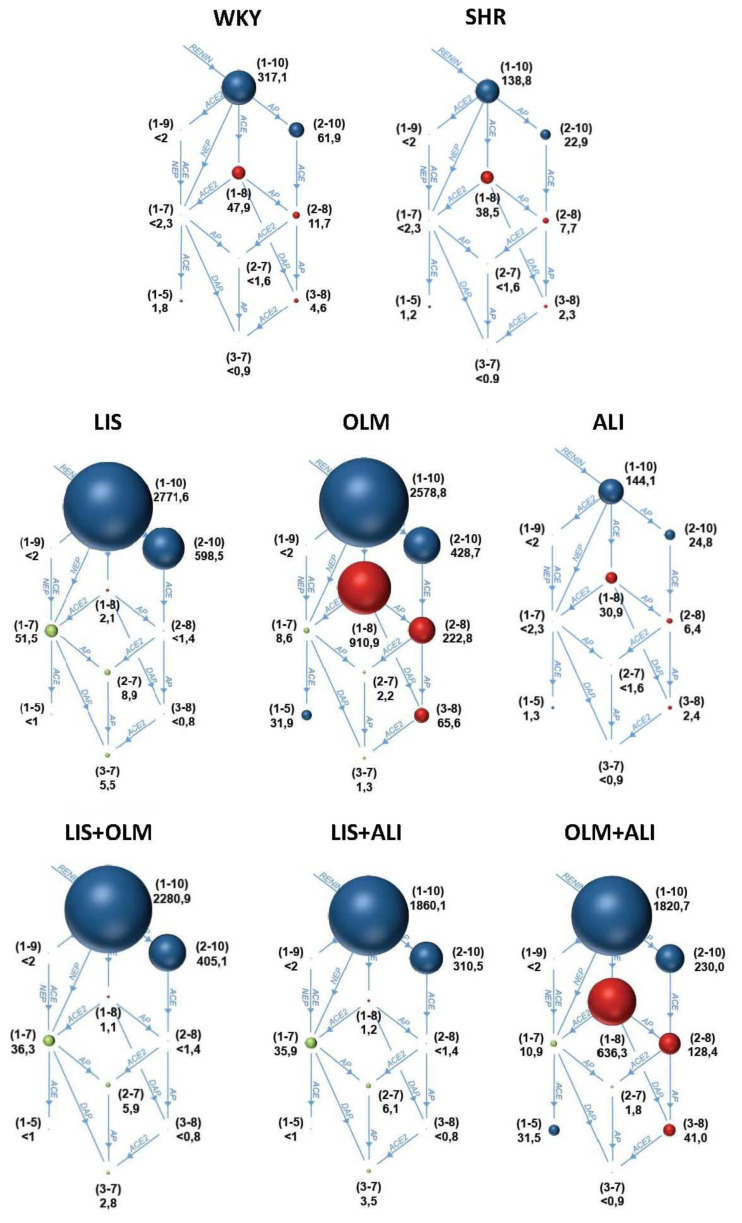
A diagram showing instantaneous (snap-shot, i.e. determined in the presence of enzyme inhibitors) angiotensin peptide levels in the serum of Wistar Kyoto rats (WKY), spontaneously hypertensive rats (SHR) and SHR-treated with 10 mg/kg/day olmesartan (OLM, n=6), 75 mg/kg/day aliskiren (ALI, n=6), or their combinations: LIS+OLM (n=5), LIS+ALI (n=5) and OLM+ALI (n=6) for 4 weeks. Nr. In brackets indicate the respective angiotensin peptide (e.g (1–10) indicates Ang I and (1–8) indicates Ang II); numbers below indicate concentration in pg/ml; AP, aminopeptidase, ACE, angiotensin-converting enzyme; NEP, neprilysisn; DAP, dual aminipeptidase.

**Fig. 2:**
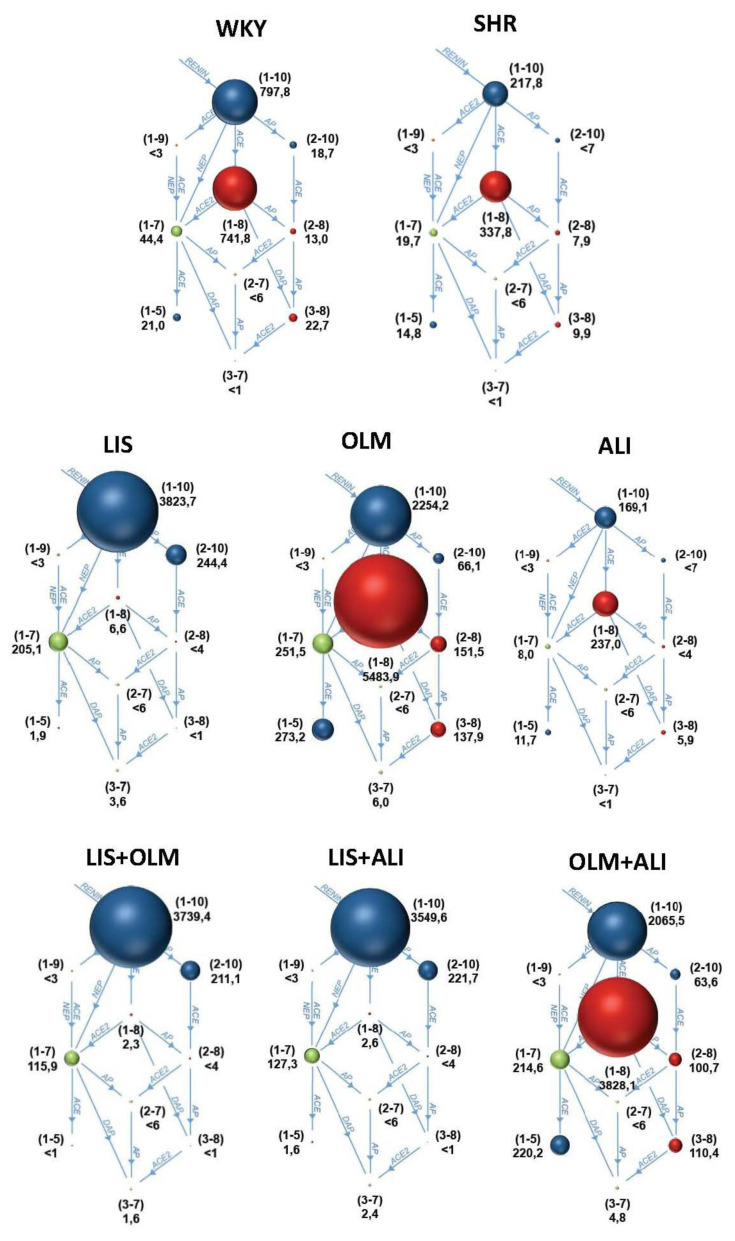
A diagram showing equilibrated (i.e. without the presence of inhibitors and after 30 min incubation at 37 °C to allow enzymatic activity) angiotensin peptide levels in the serum of Wistar Kyoto rats (WKY), spontaneously hypertensive rats (SHR) and SHR-treated with 10 mg/kg/day olmesartan (OLM, n=6), 75 mg/kg/day aliskiren (ALI, n=6), or their combinations: LIS+OLM (n=5), LIS+ALI (n=5) and OLM+ALI (n=6) for 4 weeks. Nr. In brackets indicate the respective angiotensin peptide (e.g (1–10) indicates Ang I and (1–8) indicates Ang II); numbers below indicate concentration in pg/ml; AP, aminopeptidase, ACE, angiotensin-converting enzyme; NEP, neprilysin; DAP, dual aminipeptidase.

Untreated SHR exhibited lower instantaneous Ang I, Ang II, and Ang 1–7 levels compared to WKY (Fig. 1). The equilibrium concentrations of these peptides indicated similar levels of ACE and ACE2 surrogates suggesting a downregulation of RAS in SHR on the level of renin activity (Fig. 2).

Lisinopril almost completely inhibited the ACE activity, resulting in extremely increased Ang I levels and completely suppressed Ang II levels. The Ang 1–7 levels were increased producing increase ACE2 surrogate that in this case reflects increased neprilysin (NEP) activity bypassing the Ang II. Olmesartan resulted in extremely elevated Ang I levels due to disinhibited renin feedback. However, the ACE activity was increased as well, resulting in extremely high Ang II levels and numerically increased Ang 1–7 levels (however, significantly lower compared to LIS). Aliskiren did not affect snap-shot Ang I levels, but Ang II was numerically reduced. The equilibrium analysis suggests that reduced renin activity in ALI is associated with slightly reduced ACE activity. The snapshot levels of Ang 1–7 remained unaffected while the equilibrium levels of Ang 1–7 were numerically lower in ALI compared to SHR (Table 3).

Both lisinopril combinations were characterized by suppressed ACE activity. The addition of olmesartan to lisinopril did not affect the Ang I and Ang II levels, yet it numerically reduced the Ang 1–7 levels compared to LIS. Compared to OLM, the LIS+OLM combination significantly increased the snap-shot levels of Ang 1–7, but reduced the equilibrium levels of Ang 1–7 suggesting reduced by-pass observed in the LIS group. The addition of aliskiren to lisnopril resulted in reduced Ang I levels due to renin inhibition and further reduction in both Ang II and Ang 1–7 compared to LIS. However, compared to ALI, the Ang 1–7 levels were increased due to NEP-mediated Ang 1–7 generation. The addition of aliskiren to olmesartan resulted in reduced Ang I levels due to renin inhibition and further reduction in Ang II compared to OLM. The Ang 1–7 equilibrium generation was increased compared to ALI, yet was numerically lower compared to OLM (Table 3).

### Tissue angiotensin profile

In the LV, SHR were characterized by numerically reduced Ang I and significantly reduced Ang II equilibrium levels compared to WKY. In both, LIS and OLM, the Ang I levels were increased vs. SHR suggesting a feedback mechanism. However, the Ang II levels remained lower vs. WKY in both LIS and OLM, with reduced ACE surrogate in the LV tissue. Aliskiren did not affect Ang I levels, but numerically increased Ang II levels resulting in increased ACE surrogate in the LV. The addition of both olmesartan and aliskiren to lisinopril reduced the Ang I levels compared to LIS, while the Ang II levels remained low. Addition of aliskiren to olmesartan numerically reduced Ang I levels compared to OLM, while the Ang II levels were only slightly altered being numerically between ALI and OLM. There were no significant differences in the Ang 1–7 levels in the left ventricle (Table 4).

In the kidney, SHR were characterized by numerically reduced Ang I and Ang II equilibrium levels compared to WKY. However, the ratio between Ang II and Ang I suggested increased Ang II generation in the renal tissue. In both, LIS and OLM, the Ang I levels were increased by the feedback mechanism, in the OLM group significantly. Ang II levels were lower compared to WKY and SHR in both LIS and OLM, with strong suppression in LIS. The ACE and ACE2 surrogates indicate that while in LIS the inhibited ACE was bypassed by NEP, in OLM the Ang II was exposed to increased conversion to Ang 1–7 by the ACE2. Aliskiren vs. SHR only numerically reduced Ang I levels with almost no effect on Ang II or Ang 1–7 levels. The addition of both olmesartan and aliskiren reduced the Ang I levels compared to LIS, while the Ang II levels remained low and Ang 1–7 was numerically even more reduced. The addition of aliskiren to olmesartan significantly reduced Ang I levels and numerically reduced Ang II levels compared to OLM. Unlike in the OLM group, the Ang 1–7 levels in the kidney remained unchanged vs. SHR in the OLM+ALI group (Table 4).

## Discussion

In our present study, compared to WKY, the SHR exhibited the expected hypertensive phenotype with higher SBP and myocardial hypertrophy. The magnitude of BP reduction differed substantially across treatment groups, revealing clear pharmacodynamic differences within RAS-targeting drugs. Aliskiren alone induced only a minimal SBP decrease, while olmesartan, lisinopril and the OLM+ALI combination produced pronounced and largely comparable antihypertensive effects. The strongest BP reductions, however, occurred in both lisinopril-based combinations (LIS+OLM and LIS+ALI), consistent with the advantage of blocking the cascade at multiple levels. These findings parallel some previous comparative studies in SHR demonstrating that ACE inhibitors typically provide the greater antihypertensive response than ARBs [17], while other studies have indicated comparative antihypertive action between ACEi and ARBs [18] or with RI [19]. Previous investigation on the combination of antihypertensive regimens, have demonstrated additive blood-pressure lowering when ACE inhibition is combined with ARB [18,20]. In line with the low antihypertensive effects of aliskiren were the low antihypertensive effects of the OLM+ALI combination in our experiment. The divergence between the strong BP response to ACEi-based combinations and the limited effect of OLM+ALI in our experiment further underscores the functional constraints of renin inhibition in adult SHR.

An interesting methodological aspect in our paper is the assessment of myocardial structure using various means. Assessment of structural cardiac remodeling using complementary methods revealed additional mechanistic insights. Measured HW/TL closely matched the overall pattern observed with histomorphometrically derived LVM/TL. However, the latter displayed substantially greater variability, yielding fewer statistically significant differences. This discrepancy is expected because histomorphometry of non-perfused hearts tends to underestimate cavity dimensions and inflate relative wall thickness, a limitation well documented in comparative pathology studies [21,22]. Despite these methodological constraints, histology revealed the anticipated concentric hypertrophy in SHR. Treatment-induced hypertrophy regression closely paralleled BP lowering, suggesting that structural remodeling in this model remains strongly dependent on afterload. Lisinopril, particularly in combinations, produced the most robust antihypertrophic effect, whereas aliskiren – either alone or combined – had negligible impact. In previous experiments on SHR, ACEi, ARB and direct RI each regress left-ventricular hypertrophy, with ACEi producing substantial reductions in pressure and LV mass [23], ARBs achieving significant but more modest LVM regression [24], and aliskiren reducing cardiomyocyte hypertrophy to a degree comparable to ACEi/ARB at equi-hypotensive doses [19]. Data for aliskiren in combination with ACEi or ARB are sparse, but the combined ACE inhibition and AT_1_-blockade yielded greater reductions in arterial pressure and LVM than either monotherapy [18] comparably to our experiments.

A notable methodological inconsistency emerged when comparing histomorphometry with echocardiography. While histology indicated reduced LVID in SHR, echocardiography did not confirm this finding. This discrepancy reflects the fundamental difference in physiological state: echocardiography captures the pressurized, beating heart, whereas histological measurements are obtained from a flaccid, non-perfused organ. Previous work has shown that lack of perfusion fixation causes passive ventricular collapse, artificially lowering LVID and exaggerating relative wall thickness [13,14]. Nevertheless, the echocardiographic LVM/TL pattern qualitatively mirrored the structural findings, with the greatest reductions again observed in the lisinopril combinations, albeit with higher variability.

Although treatment groups differed in several echocardiographic functional parameters, the post-hoc analysis did not identify statistically significant differences. SHR displayed only mild tendencies toward systolic (SV, FS) and diastolic (EDV) dysfunction. The phenotype observed in our study is determined by the age of SHR employed. Some studies indicate a progressive increase of LV hypertrophy from four weeks of age with systolic (fractional shortening, mid-wall shortening) and diastolic (E/A ratio, deceleration time) dysfunction emerging between 8 and 16 weeks [22]. The decline in LV function in SHR has been reported to be reversible with fosinopril [25], losartan [26] or in ischemia–reperfusion in SHR and double-transgenic renin–angiotensin rats with aliskiren as well [27,28].

One of the most distinctive contributions of this study is the comprehensive characterization of systemic and tissue RAS activity across treatment regimens. Moreover, for serum levels we report both, the snapshot and equilibrium peptide levels. Snapshot measurements capture the peptide concentrations at the moment of sampling with proteases inhibited, whereas equilibrium measurements allow endogenous enzymes (ACE2, NEP, PEP) to reshape the peptide pool over time [29]. We report reduced renin activity in SHR, reflected in lower circulating Ang I and Ang II levels compared to WKY, indicating a proximal substrate limitation within the cascade. The available studies are inconsistent regarding whether SHR represent a low or high renin model. In fact, some studies report an age-dependent transition from low to high renin model [30,31], other studies report the opposite development [32,33]. Our study using a reliable LS/MS-method consistently in serum as well as the tissues confirms that SHR at the age of 16 weeks represent a low renin model. This likely explains the modest antihypertensive effect of aliskiren.

RAS peptide profiling revealed distinctly different pharmacodynamic patterns across treatments. Both lisinopril and olmesartan markedly increased Ang I, in line with the classical AT1-mediated feedback stimulation of renin release [11,34,35] and accumulation of upstream substrate when distal conversion or receptor signaling is blocked. Olmesartan, as expected, also increased Ang II through unopposed ACE activity. Both drugs elevated Ang 1–7 levels, but through mechanistically distinct routes: lisinopril enhanced ACE-independent Ang 1–7 formation, most likely via NEP – the alternative route of Ang I degradation [11,34], whereas olmesartan increased Ang 1–7 through heightened ACE activity. The later has been previously suggested for the left ventricle of normotensive rats [36] and right ventricle in monocrotaline-induced pulmonary hypertension [37]. Here we are the first to report this putative mechanism in the serum in SHR. In contrast, aliskiren suppressed upstream substrate availability and reduced both Ang II and Ang 1–7, consistent with proximal inhibition limiting downstream peptide formation. Because Ang 1–7 can be generated via multiple pathways (ACE2, NEP) [11], the net effect of each treatment reflects the interplay between substrate availability and these competing enzymatic routes.

A particularly relevant observation is that the lisinopril-based combinations displayed lower circulating Ang 1–7 levels than either monotherapy. This indicates that the compensatory NEP-dependent Ang 1–7 formation induced by ACE inhibition alone is blunted when lisinopril is combined with either renin or AT1 blockade. Similarly, adding aliskiren to olmesartan reduced Ang I availability and consequently reduced Ang 1–7 generation. Considering that Ang 1–7 exerts vasodilatory, antifibrotic and anti-inflammatory effects [10,38], these combination-induced decreases may attenuate the cardioprotective potential observed with monotherapies. Notably, the OLM+ALI combination increased Ang 1–7 compared with ALI alone due to increased Ang II substrate availability, illustrating the complex interplay between proximal and distal RAS inhibition.

The longstanding debate concerning the relative contribution of circulating versus tissue RAS provides a broader context for interpreting our findings. While some studies emphasize the predominance of the circulating system [39,40,41] others highlight robust local RAS activity, particularly in the kidneys [9,42,43] and the central nervous system [43]. Our results support a hybrid model: SHR displayed a low-RAS phenotype in both plasma and LV tissue, whereas kidney RAS activity remained comparatively preserved. This observation aligns with evidence demonstrating high ACE and ACE2 expression in the kidney in particular in younger SHR [45].

Among the most striking findings was the markedly reduced LV Ang II concentration in olmesartan-treated SHR, despite extremely high circulating Ang II levels. This phenomenon is best explained by AT1-receptor-dependent myocardial uptake of circulating Ang II. AT1 blockade prevents sequestration of Ang II, producing very low LV levels even when systemic levels rise sharply corroborating previous similar results in the cardiac [40] or the brain tissue of SHR [41]. Given the very low intrinsic ACE activity in LV tissue, our findings strongly support the concept that most LV Ang II originates from the circulation rather than local synthesis. In contrast to the LV, the kidney Ang II levels in olmesartan-treated animals are increased compared to the LV of these rats yet, decreased, in regard to untreated SHR and the high Ang I levels. Moreover, the kidneys of olmesartan-treated rats show high Ang 1–7 levels. Together with elevated renal Ang 1–7 this profile suggests either (i) higher local ACE activity that in the presence of AT1 receptor blockade increases Ang II substrate availability for conversion to Ang 1–7 or (ii) greater renal AT2 receptor abundance, providing binding sites for Ang II and Ang 1–7 irrespective of local peptide synthesis

Support for stronger local ACE activity in the kidney arises from the ALI group, which exhibited low circulating and LV Ang II yet preserved renal Ang II levels comparable to SHR. Conversely, the near-complete depletion of LV Ang II in all olmesartan-containing groups underscores the indispensable role of AT1 receptors in retaining Ang II in cardiac tissue and limiting its downstream metabolism.

When interpreting the tissue Ang 1–7 levels, we need to bear in mind that the ACE2 surrogate reflects contributions from both membrane-bound and soluble enzyme.

Furthermore, the downstream metabolism of angiotensin peptides including the action of aminopeptidase A (APA) and N (APN) activities [2,11] should be considered. Our results could suggest increased angiotensin peptide degradation in the LV and reduced in the kidney.

Finally, tissue analysis revealed that combinations containing lisinopril consistently reduced renal Ang 1–7 relative to monotherapies, and the OLM+ALI combination also lowered Ang 1–7 compared to OLM alone. Because renal Ang 1–7 plays a significant protective role — limiting fibrosis and improving renal hemodynamics [2,10,11] — these reductions may be physiologically meaningful and could partially explain the lack of clinical benefit observed with dual RAS blockade despite greater BP lowering. Furthermore, these considerations advocate for interventions combining the blockade of the deleterious arm of RAS with the stimulation of the protective RAS axis [46]

## Conclusions

Our study on dual RAS blockade in SHR shows that ACEi produced the most pronounced antihypertensive and antihypertrophic effects, with additional benefit when combined with an ARB or a RI. In contrast, RI – either alone or in combination – had only modest effects in this low-renin strain, underscoring the strong dependence of RI efficacy on baseline renin status. The morphological changes of the myocardium largely mirrored the blood pressure responses across treatment groups, reinforcing the hemodynamic basis of structural remodeling in SHR.

Further, our results demonstrate that the circulating RAS strongly shapes the distribution of peripheral RAS peptides, particularly in the left ventricle, where Ang II levels appear to reflect uptake of circulating peptides rather than local synthesis. In the kidney, however, the peptide profile suggests a greater contribution of local enzymatic activity, consistent with higher intrinsic ACE/ACE2 expression in this organ.

Finally, we show that most dual RAS blockade combinations reduce circulating and renal Ang 1–7 levels relative to monotherapies, potentially attenuating the protective Ang 1–7/Mas axis despite more effective blood pressure lowering. This mechanism may help explain why clinical trials of dual RAS inhibition failed to demonstrate improved outcomes despite enhanced BP reduction.

Collectively, these results advocate a cautious, mechanism-aware approach to combining RAS inhibitors and provide a rationale for therapeutic strategies that balance hemodynamic control with preservation of the Ang 1–7 axis.

## Figures and Tables

**Table 1:** Basic physiological and histological characteristics

n	WKY	SHR	LIS	OLM	ALI	LIS+OLM	LIS+ALI	OLM+ALI
	5	6	5	6	6	5	5	6
*SBP (mmHg)* *	124.4±3.8	188.6±3.1^w^	134.5±11.2^s^	145.7±4.2^s^	178.3±7.6^wl^	104.1±10.9^so^	113.9±13.3^sa^	142.9±11.9^a^
*HR (/min)* *	389.6±10.8	479.8±11.2	402.8±34.9	481.3±12.2	411.1±26.3	390.7±21.3	424.3±26.6	414.9±31.4
*HW (mg)* *	862.4±30.6	1027.5±13.2^w^	855.4±33.5	971.8±41.4	1096.0±37.8^wl^	769.0±35.4^so^	764.4±59.7^sa^	951.0±22.0
*TL (mm)* *	41.2±0.2	39.2±0.2^w^	39.5±0.1^w^	39.5±0.3	39.5±0.2^w^	39.3±0.2^w^	39.1±0.3^w^	39.3±0.3^w^
*HW/TL (mg/mm)* *	20.9±0.7	26.2±0.4^w^	21.7±0.8^s^	24.6±0.9	27.8±1.0^w^	19.6±0.8^so^	19.5±1.4^sa^	24.2±0.5
*Histo LVID (mm)*	5.00±0.26	4.69±0.24	4.92±0.22	5.02±0.21	4.91±0.30	4.75±0.24	3.67±0.77	5.56±0.22
*Histo LVWT (mm)* *	2.79±0.10	3.12±0.07	2.71±0.04	2.79±0.08	2.75±0.07	2.55±0.15^s^	2.44±0.05	2.69±0.10
*Histo LVM (mg)* *	942.7±102.6	985.4±50.7	839.1±60.7	967.0±82.1	849.0±105.0	723.1±65.8	587.9±55.2	968.0±77.4
*Histo LVM/TL (mg/mm)* *	22.9±2.6	25.1±1.2	21.3±1.6	24.6±2.1	21.4±2.6	18.4±1.7	15.0±1.4	24.5±2.0

Values are mean ± standard error of mean;

* p<0.05 one-way ANOVA with Tukey post-hoc test (selected comparisons, significance indicated against WKY (^w^), SHR (^s^), LIS (^l^), OLM (^o^), ALI (^a^));

n, number; SBP, systolic blood pressure; HR, heart rate; HW, heart weight; TL, tibia length; values determined by histomorphometry: LVID, left ventricle inner diameter; LVWT, left ventricle wall thickness; LVM, left ventricle mass (spherical assumption).

**Table 2:** Echocardiography characteristics

	WKY	SHR	LIS	OLM	ALI	LIS+OLM	LIS+ALI	OLM+ALI
*LVIDs (mm)* *	4.18±0.09	4.47±0.18	4.74±0.38	4.77±0.10	4.73±0.17	4.74±0.09	4.33±0.36	5.32±0.04^w^
*LVIDd (mm)* *	7.12±0.12	7.16±0.26	7.30±0.29	7.56±0.17	7.35±0.11	7.27±0.10	6.76±0.48	8.20±0.08^ws^
*IVSs (mm)*	2.11±0.04	2.19±0.07	2.07±0.12	2.21±0.04	2.13±0.09	1.99±0.06	1.86±0.11	2.25±0.14
*IVSd (mm)*	1.24±0.08	1.27±0.08	1.21±0.03	1.23±0.06	1.22±0.07	1.09±0.04	1.06±0.12	1.27±0.05
*PWTs (mm)*	2.11±0.10	2.12±0.10	2.03±0.20	1.84±0.15	1.97±0.16	1.81±0.03	2.00±0.08	2.03±0.15
*PWTd (mm)*	1.35±0.08	1.55±0.07	1.64±0.11	1.29±0.05	1.54±0.13	1.32±0.07	1.46±0.12	1.42±0.13
*ESV (ml)* *	0.183±0.011	0.225±0.026	0.277±0.054	0.264±0.016	0.262±0.025	0.259±0.013	0.215±0.046	0.362±0.009^ws^
*EDV(ml)* *	0.408±0.019	0.421±0.041	0.443±0.048	0.482±0.030	0.446±0.017	0.431±0.016	0.370±0.066	0.601±0.016^ws^
*SV (ml)* *	0.225±0.022	0.196±0.018	0.166±0.022	0.218±0.022	0.183±0.008	0.172±0.006	0.154±0.022	0.239±0.018
*FS (%)*	41.2±1.4	37.7±0.8	35.3±3.3	37.0±1.0	35.6±1.5	34.8±0.6	36.3±1.0	35.0±0.8
*Echo LVM(mg)*	1062.0±26.6	1133.3±56.5	1150.0±37.8	1096.3±35.0	1131.7±40.5	1037.1±27.5	1015.3±50.0	1216.7±58.2
Echo LVM/TL (mg/mm)*	25.8±0.7	28.9±1.5	29.1±0.9	27.9±0.8	28.7±1.2	26.5±0.8	25.9±1.1	30.8±1.4

Values are mean ± standard error of mean;

* p<0.05 one-way ANOVA with Tukey post-hoc test (selected comparisons, significance indicated against WKY (^w^), SHR (^s^), LIS (^l^), OLM (^o^), ALI (^a^));

s/d indicates value in systole/diastole respectively; LVID, left ventricle inner diameter; IVS, interventricular septum thickness; PWT, posterior wall thickness; ESV, end-systolic volume; EDV, end-diastolic volume; SV, stroke volume; FS, fractional shortening; LVM, left ventricle mass.

**Table 3:** Angiotensin peptides analysis in the plasma

a. Snap-shot	WKY	SHR	LIS	OLM	ALI	LIS+OLM	LIS+ALI	OLM+ALI
Ang I (pg/mg)^*^	317.1±81.6	138.8±18.2	2771.6±442.5^wsa^	2578.8±453.6^wsa^	144.1±41.1	2280.9±464.4^ws^	1860.1±199.8^wsa^	1820.7±190.3^wsa^
Ang II (pg/ml)^*^	47.9±7.0	38.5±10.0	2.1±0.5	910.9±155.7wsl	30.9±9.6lo	1.1±0.5o	1.2±0.2	636.3±129.2wsa
Ang 1–7 (pg/ml)^*^	2.08±0.54	1.10±0.12	51.48±11.40^wsa^	8.59±2.01l	1.05±0.10lo	36.33±2.79wso	35.87±6.62^wsa^	10.85±2.30
	
**b. Equilibrium**	**WKY**	**SHR**	**LIS**	**OLM**	**ALI**	**LIS+OLM**	**LIS+ALI**	**OLM+ALI**
	
Ang I (pg/mg)^*^	797.8±96.9	217.8±28.4	3823.7±744.7^wsa^	2254.2±279.6	169.1±50.9^lo^	3549.6±523.2^ws^	2839.7±817.5^sa^	2065.5±235.2
Ang II (pg/ml)^*^	741.8±65.0	337.8±29.5	6.6±3.5	5483.9±828.1^wsl^	237.0±88.3^o^	2.6±1.2^o^	2.1±1.1	3828.1±522.9^wsa^
Ang 1–7 (pg/ml)^*^	44.4±5.6	19.7±2.7	205.1±31.6^wsa^	251.5±38.8^ws^	8.0±4.1^lo^	127.3±11.5^o^	101.9±27.0	214.6±34.7^wsa^
ACE surrogate (x100)^*^	101.9±20.4	175.5±34.1	0.1±0.1^s^	246.8±24.2^wsl^	152.3±25.3^l^	0.07±0.03^so^	0.1±0.0^sa^	198.3±32.4
ACE2 surrogate (x100)^*^	6.33±1.20	6.21±1.28	6967.18±3726.83	4.74±0.63	2.87±0.74	8479.51±3376.86	6783.61±3117.37	5.75±0.59

**Table 4:** Angiotensin peptide analysis in the left ventricle and kidney

Equilibrium LV	WKY	SHR	LIS	OLM	ALI	LIS+OLM	LIS+ALI	OLM+ALI
Ang I (pg/ml)*	9.41±0.64	7.79±0.74	33.42±1.44^ws^	80.17±8.12^ws^	8.53±0.45^lo^	7.73±1.00^lo^	13.39±4.21	45.79±9.16^soa^
Ang II (pg/ml)*	15.4±0.9	4.4±0.3^w^	4.8±0.2^w^	5.6±0.8^ws^	9.9±2.2	5.1±0.5^w^	5.0±0.5^w^	7.2±2.6^w^
Ang 1–7 (pg/ml)	8.41±0.55	7.26±0.67	7.56±0.24	6.73±0.27	7.33±0.37	8.16±1.15	7.43±1.03	7.37±0.47
ACE surrogate (x100)*	165.7±13.1	37.4±9.9^w^	12.4±2.2^w^	15.3±7.8^ws^	122.4±31.0^slo^	62.4±0.5^w^	37.7±10.4^wa^	16.7±4.4^wa^
ACE2 surrogate (x100)*	55.1±3.5	163.5±5.7^w^	156.6±1.5^wo^	131.8±16.5^w^	100.8±24.6^ol^	159.4±6.5^wo^	147.0±9.0^wa^	137.8±20.0^wa^

**Equilibrium Kidney**	**WKY**	**SHR**	**LIS**	**OLM**	**ALI**	**LIS+OLM**	**LIS+ALI**	**OLM+ALI**

Ang I (pg/ml)*	522.8±94.3	254.5±18.9	467.0±83.2^o^	1048.5±144.9^ws^	193.2±52.3^ol^	168.7±37.6^o^	175.0±12.1	524.6±103.3^o^
Ang II (pg/ml)*	271.6±27.3	228.6±34.4	18.0±6.1^ws^	127.3±8.0^wsl^	238.4±31.8^l^	5.8±0.3^wso^	6.0±0.5^wsa^	83.9±9.6^wsa^
Ang 1–7 (pg/ml)*	86.4±43.0	30.2±3.1	64.7±33.5	90.3±20.5	30.4±14.8	17.4±3.7	14.3±5.1	39.4±17.8
ACE surrogate (x100)	58.6±11.9	94.5±19.3	3.6±0.8^sa^	13.6±2.3^sa^	145.2±19.8^ws^	4.5±1.2^s^	3.5±0.4^wsa^	18.2±3.2^sa^
ACE2 surrogate (x100)*	30.0±14.4	14.6±2.9	362.6±118.8^ws^	74.4±19.8^l^	12.5±4.6^lo^	298.8±63.8^ws^	231.0±74.0	45.3±17.7

Values are mean ± standard error of mean;

* p<0.05 one-way ANOVA with Tukey post-hoc test (selected comparisons, significance indicated against WKY (^w^), SHR (^s^), LIS (^l^), OLM (^o^), ALI (^a^)); all tissue values are equlibrium peptide levels, levls without the presence of inhibitors and after 30 min incubation at 37 °C to allow enzymatic activity;

ACE, angiotensin converting enzyme surrogate based on Ang II/Ang I ratio; ACE2, angiotensin converting enzyme 2 surrogate based on Ang 1–7/Ang II ratio
